# Evaluation of platelet lysate as a substitute for FBS in explant and enzymatic isolation methods of human umbilical cord MSCs

**DOI:** 10.1038/s41598-018-30772-4

**Published:** 2018-08-20

**Authors:** Sangeetha Kandoi, Praveen kumar L, Bamadeb Patra, Prasanna Vidyasekar, Divya Sivanesan, Vijayalakshmi S., Rajagopal K., Rama Shanker Verma

**Affiliations:** 1 Indian Institute of Technology Madras, Bhupat and Jyoti Mehta School of Biosciences, Department of Biotechnology, Chennai, 600036 India; 2 Vels University, Department of Biotechnology, Chennai, 600117 India; 3 Ramakrishna Mission Vivekananda College, Department of Plant Biology and Biotechnology, Chennai, 600004 India

## Abstract

Mesenchymal stem cells (MSCs) have immense potential for cell-based therapy of acute and chronic pathological conditions. MSC transplantation for cell-based therapy requires a substantial number of cells in the range of 0.5–2.5 × 10^6^ cells/kg body weight of an individual. A prolific source of MSCs followed by *in vitro* propagation is therefore an absolute prerequisite for clinical applications. Umbilical cord tissue (UCT) is an abundantly available prolific source of MSC that are fetal in nature and have higher potential for *ex-vivo* expansion. However, the *ex-vivo* expansion of MSCs using a xenogeneic supplement such as fetal bovine serum (FBS) carries the risk of transmission of zoonotic infections and immunological reactions. We used platelet lysate (PL) as a xeno-free, allogeneic replacement for FBS and compared the biological and functional characteristics of MSC processed and expanded with PL and FBS by explant and enzymatic method. UCT-MSCs expanded using PL displayed typical immunophenotype, plasticity, immunomodulatory property and chromosomal stability. PL supplementation also showed 2-fold increase in MSC yield from explant culture with improved immunomodulatory activity as compared to enzymatically dissociated cultures. In conclusion, PL from expired platelets is a viable alternative to FBS for generating clinically relevant numbers of MSC from explant cultures over enzymatic method.

## Introduction

Mesenchymal stem cells (MSCs) have acquired a prominent role in cell therapy strategies as they encompass pro-angiogenic^1^, anti-apoptotic^2^, anti-fibrotic^3^, anti-inflammatory^4^ and tissue regenerative properties^5^. Furthermore, MSCs are unhindered by concerns of graft versus host disease (GVHD), formation of teratomas^6^, and ethical concerns^7^. The therapeutic potential and appeal of MSC is evident from the increasing number of ongoing and planned clinical trials sponsored by various governments, academics and industry. A total of 656 clinical trials have been registered with www.clinicaltrials.gov as of February 2018 with a majority of them demonstrating the safety and efficacy of MSCs for clinical use. The recommended dosage of MSC for transplantation per infusion varies from 0.5–2.5 × 10^6^ cells/kg body weight of the individual^8^. Obtaining such high yields of autologous and allogeneic clinical grade MSCs for clinical application is directly linked to the source, quality of culture conditions, media, and growth supplements under which they are expanded.

Fetal bovine serum (FBS) is the standard growth supplement used in animal cell culture for maintenance and propagation of various cell lines *in vitro* and over 80% of clinical trials use MSCs that have been expanded with FBS as growth supplement^9,10^. However FBS carries the risk of transmission of known and unknown pathogens and elicits immunological reactions^11^. FBS also suffers from batch-to-batch variability, displays heterogeneous cytokine profile and contains xenogeneic bovine proteins which are known to get internalized into the cells during expansion^12^. Importantly, its production raises several ethical issues over animal welfare^13^. The use of an allogeneic human sourced replacement such as human umbilical cord blood serum (UCBS)^14^ and peripheral blood components such as serum, plasma fraction^15^, platelet rich plasma (PRP) and platelets^16^, alleviates these concerns. UCBS has been shown to have an enhanced effect on MSC culture and differentiation in our previous study^17^, however, the production of UCBS is hampered by the limited volume of yield.

**Figure 1:**
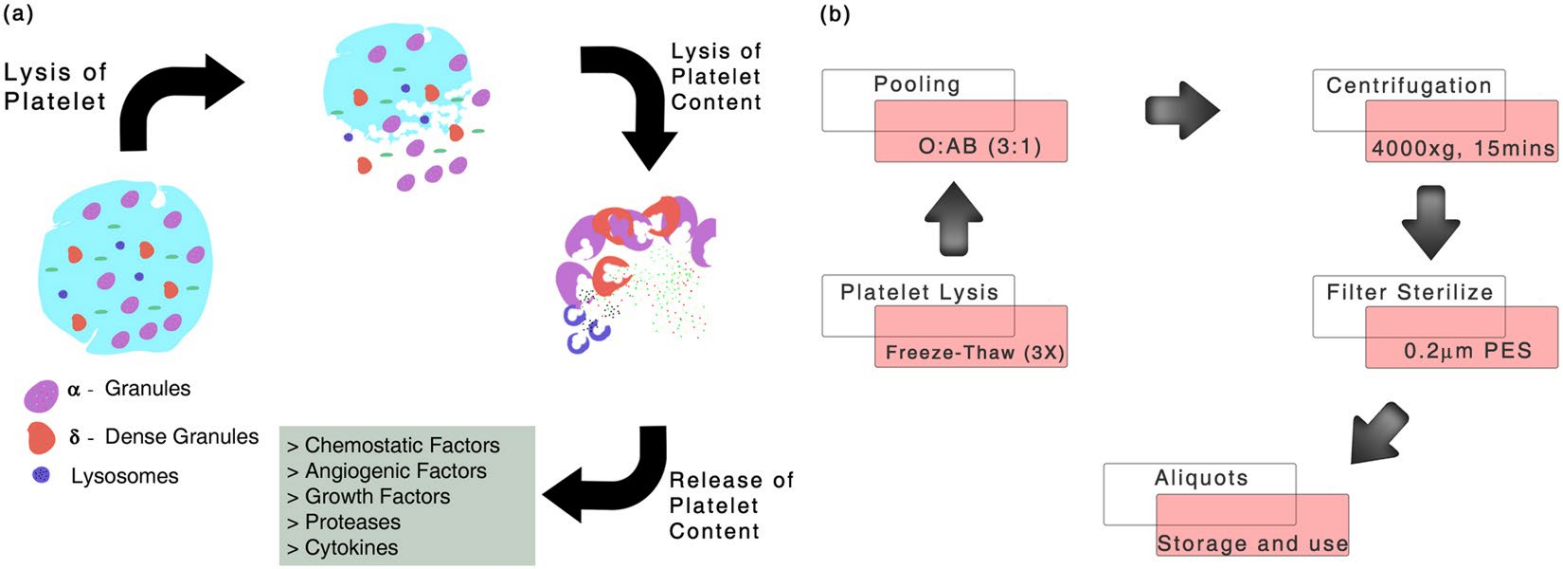
Platelet content and preparation of pooled human platelet lysate (pHPL). (**a**) Illustration showing the release of platelet granule contents which include cytokines, angiogenic factors, proteases and chemostatic factors on lysis (**b**) Overview of freeze-thaw mechanism of platelet lysate preparation, pooling and long-term storage.

Peripheral blood components such as platelets have a shelf life of 5 days following which there is an increased risk of platelet aggregation along with the risk of acquiring bacterial contamination due to their higher storage temperature of 2–8 °C making them unsafe for transfusion^18^. Blood banks then proceed to classify and discard these expired platelet units as biological waste^19^. Platelets store abundant growth factors, cytokines and other regulatory molecules within platelet granules that are released by thrombin activation and clotting^20^. Platelet lysis through freeze-thaw, sonication or chemical treatment disrupts the platelet membrane and can efficiently release these factors *in vitro* (Fig. 1a). Studies have already employed the use of MSC obtained from multiple origins by *ex-vivo* expansion with PL^21,22^ for treating knee cartilage injuries, GVHD, osteonecrosis of femoral head and aneurysmal bone cyst (ABC)^23,24^.

In this study, we show that platelet lysate (PL) prepared by several freeze-thaw cycles (Fig. 1b), can be used for harvesting and propagating clinical grade MSC from human umbilical cord tissue (hUCT). Uniquely, human platelet lysate (hPL) was employed as a supplement for isolation of MSCs from hUCT via explant and enzymatic dissociation strategies. Expanded MSCs were further characterized as per International Society for Cellular Therapy (ISCT) standards and an *in vitro* immunocyte suppression assay was performed to assess functional applicability in allogeneic transplantation. This study aims to demonstrate the feasibility of PL as a potent replacement for FBS in the culture supplement with none of the drawbacks associated with FBS and UCBS.

## Results

### PL supports isolation and expansion of MSC from hUCT

**Figure 2:**
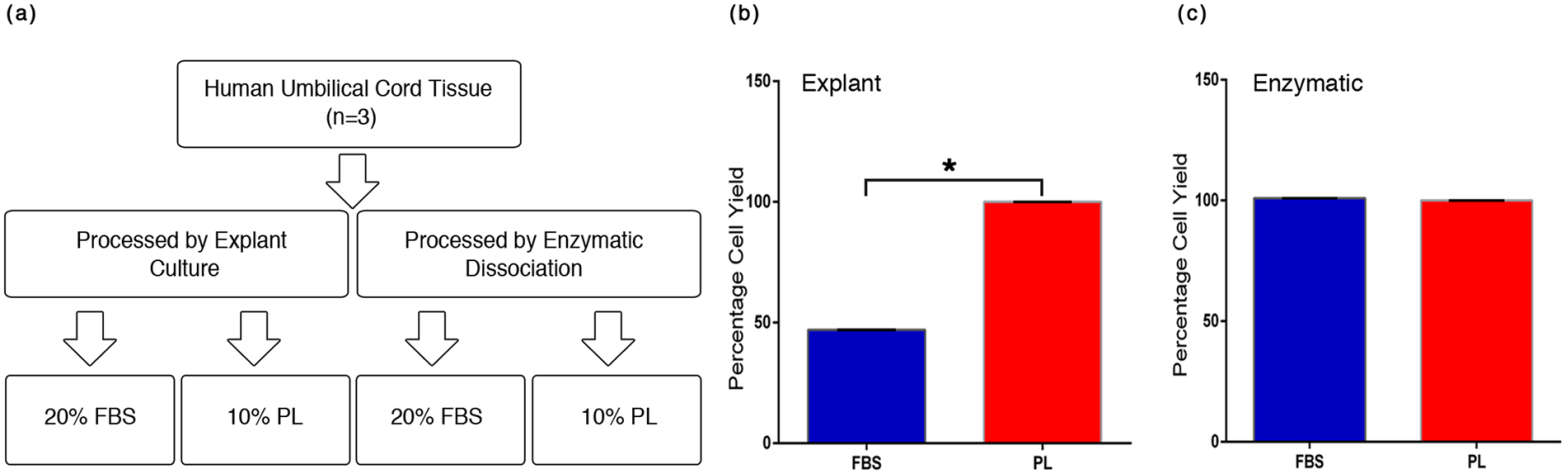
Umbilical cord tissue processing and MSC yield. (**a**) A schematic representation for isolation of MSC from hUCT using explant and enzymatic dissociation culture employing 20% FBS and 10% PL supplemented medium. (**b**) Comparison of percentage of viable cell yields from explant and (**c**) enzymatically dissociated cultures supplemented with 20% FBS and 10% PL using trypan blue dye exclusion. Results are expressed as mean ± SEM of three processed samples. Paired one tailed student’s t test for explant cultures and enzymatic cultures were P = 0.03 and P = 0.49 respectively. *P < 0.05.

Umbilical cord tissue is routinely processed by explant culture or enzymatic dissociation. To validate the ability of PL to support the proliferation and expansion of MSC isolated by these two methods, primary cultures were supplemented with 10% PL and 20% FBS respectively (Fig. 2a). Explant cultures supplemented with 10% PL showed a 2-fold increase in viable cell number over 20% FBS supplemented cultures (Fig. 2b). No significant difference in viable cell number was observed in enzymatically dissociated cultures supplemented with PL and FBS (Fig. 2c). We inferred that PL could be advantageous and efficient in harvesting significant numbers of viable cells from explant cultures where the original microenvironment is maintained.

### PL supplemented cultures meet ISCT standards of MSC characterization

**Figure 3:**
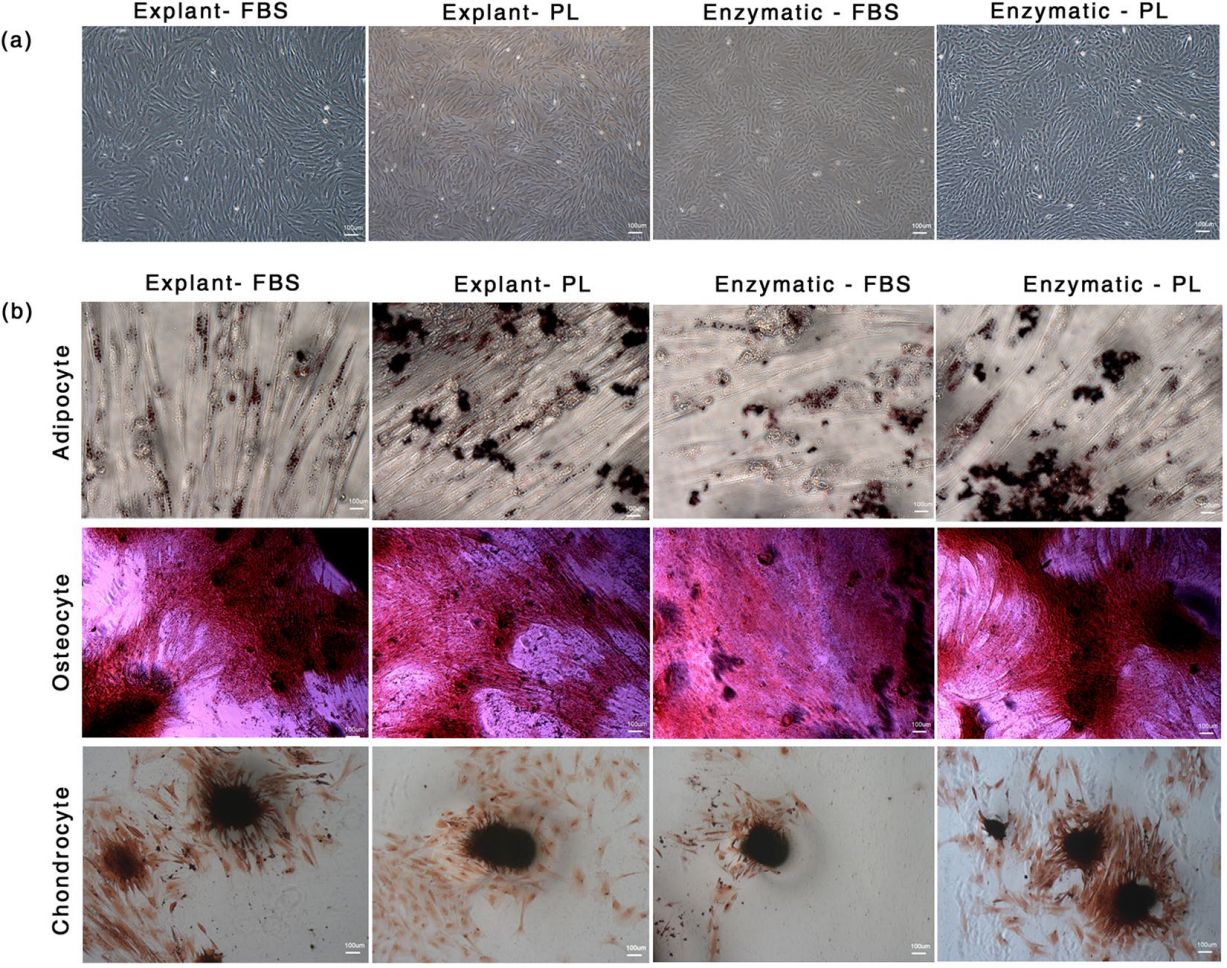
MSC characteristics as per ISCT criteria. (**a**) Plastic adherent MSC with fibroblast like shape. (**b**) Tri-lineage differentiation potential as shown by oil red O, alizarin red S and safranin O respectively for adipocytes, osteocytes and chondrocytes.

**Figure 4:**
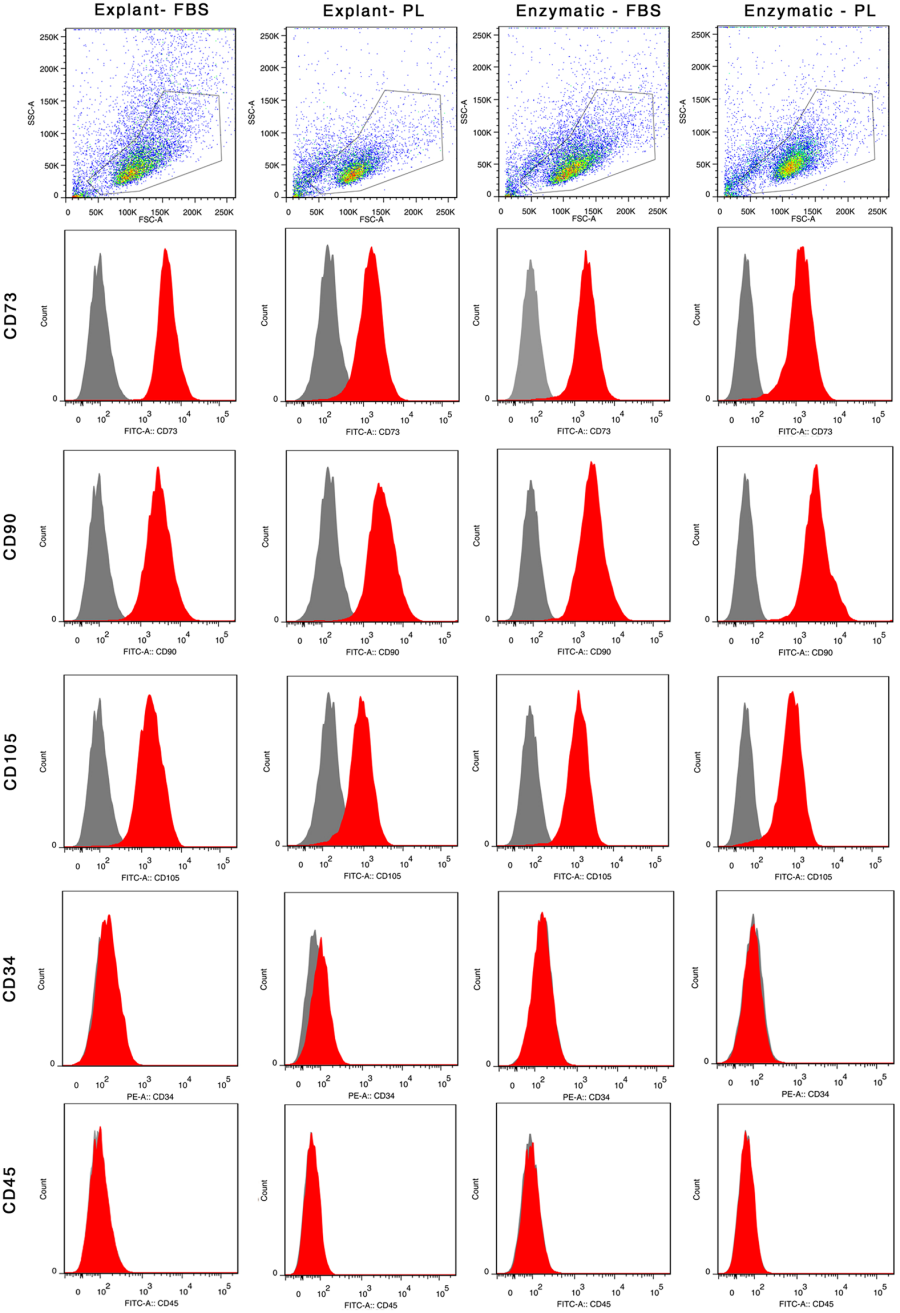
Immunophenotypic analysis by flow cytometry. Representative image of flow cytometry analysis of UC-MSC at passage 2 probed with conjugated antibodies against MSC and hematopoietic markers CD73, CD90, CD105, and CD34, CD45 respectively.

The ISCT has defined criteria for characterization of MSCs which includes adherence to plastic surface, expression of specific surface markers and tri-lineage differentiation potential. Primary cultures of hUCT-MSC supplemented with 20% FBS and 10% PL showed spindle-shape and fibroblast like morphology that was maintained over long-term culture (Fig. 3a). Flow cytometry analysis for CD73, CD90 and CD105 surface marker expression revealed positive populations in both PL and FBS supplemented cultures that were also negative for hematopoietic lineages such as CD34 and CD45 (Fig. 4, Table 1). PL and FBS supplemented cultures exhibited equal potential for differentiation into adipocyte, osteocyte and chondrocyte lineage (Fig. 3b). AIM stimulated the production of large, flattened, oval cells with lipid droplet accumulation that were positive for oil red O staining after 14 days while control cells (uninduced) remained spindle shaped with no accumulation of lipid droplets (Fig. 3b). OIM stimulated the MSC cultures to produce typical morphological features of osteocytes with mineralization of calcium deposits that were stained positive for alizarin red S while control cells (uninduced) remained spindle shaped with no calcium deposits (Fig. 3b). Similarly, CIM differentiated MSCs condensed into nodule-like structures with a high intensity of glycosaminoglycan production from such cell structures as revealed by safranin O staining (Fig. 3b). No such micromass formation was observed in control MSCs. These results show that PL cultures satisfy all ISCT criteria for MSC cultures equivalent to the prevailing standard supplement: FBS.

**Table 1:** Surface marker expression of umbilical cord tissue derived mesenchymal stem cells cultured in FBS and PL supplements assessed by flow cytometric analysis at P2.

Surface Marker (%)	CD73	CD90	CD105	CD34	CD45
Explant FBS	97.0 ± 1.1	95.1 ± 1.7	96.6 ± 1.3	0.51 ± 0.3	0.78 ± 0.5
Explant PL	91.6 ± 3.7	90.9 ± 4.5	91.2 ± 1.0	0.18 ± 0.1	0.12 ± 0.0
Enzymatic FBS	96.1 ± 0.8	94.8 ± 2.0	96.1 ± 1.9	0.79 ± 0.3	0.52 ± 0.1
Enzymatic PL	97.7 ± 0.6	95.1 ± 2.6	98.3 ± 0.3	0.18 ± 0.1	0.19 ± 0.1

Data presented as mean percentage ± SEM of three independent experiments. Statistical comparisons were made within explant and enzymatic groups respectively. There was no statistical significant difference noted within the groups.

### PL supplementation does not alter cell cycle dynamics of MSC

**Figure 5:**
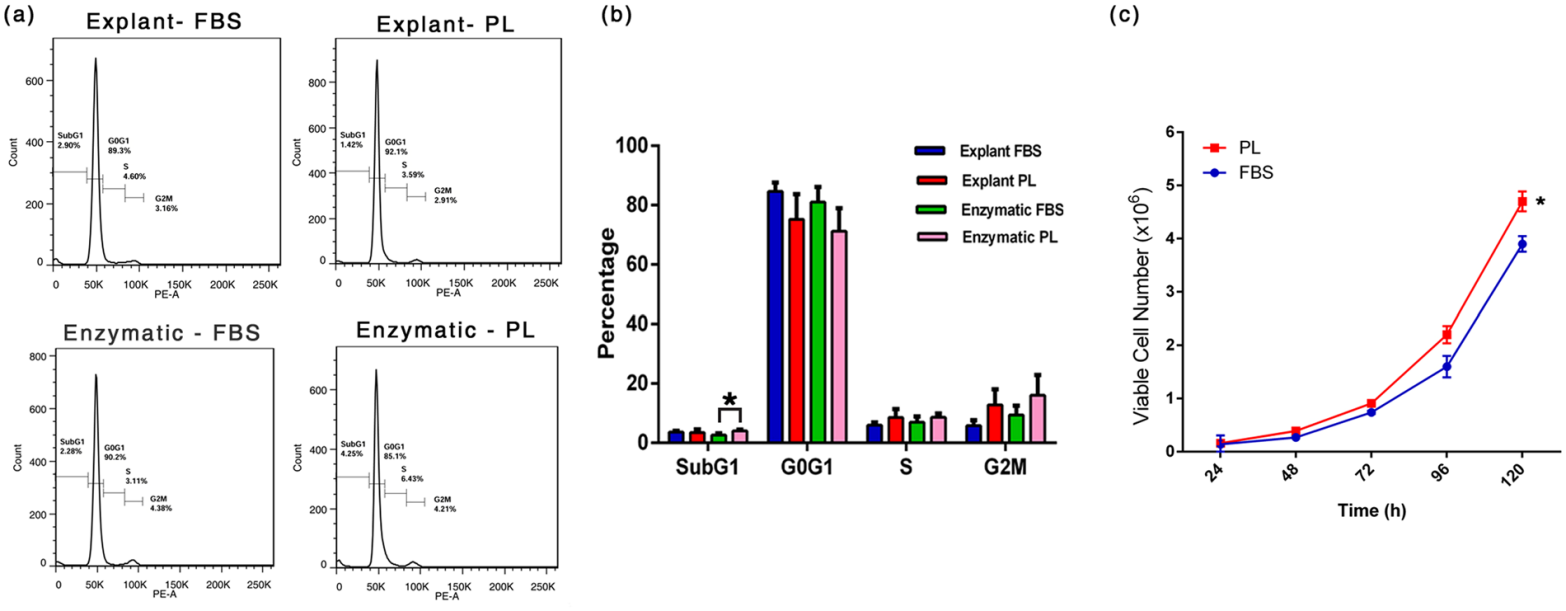
Cell cycle analysis and growth kinetics. (**a**) Representative histograms of cell cycle in synchronized MSCs (**b**) DNA contents according to the cell cycle phase G0/G1 phase, S phase and G2/M phase for PL and FBS supplemented MSC cultures. Data are represented as mean of three independent experiments. Paired one tailed student’s t test for SubG1 phase of enzymatic cultures was P = 0.02. (**c**) Growth rate of UCT-MSC cultured in PL was significantly higher than those in FBS on day 5 by trypan blue dye exclusion (P = 0.05). *P ≤ 0.05.

To determine whether PL influences cell cycle dynamics of explant and enzymatically derived MSCs differently with regard to FBS supplementation, we analyzed the percentage of cells at each phase of the cell cycle using propidium iodide by flow cytometry. No significant difference was observed among PL and FBS supplementation with 75–85% and 71–81% of cells residing in G0/G1 phase of explant and enzymatically derived MSCs respectively (Fig. 5a,b, Table 2). A population doubling time (PDT) assay was used to assess any difference in growth kinetics of explant culture derived MSCs in PL and FBS supplements. The PDT of PL and FBS supplemented cultures of MSC was 20.95 h and 22.25 h respectively indicating a significantly faster cell growth (P ≤ 0.05, using paired one tailed T-Test) of UCT-MSC for PL cultures (Fig. 5c).

**Table 2:** Cell cycle studies representing the percentage of cells in phases: SubG1 phase, G0G1 phase, S phase and G2/M phase for umbilical cord tissue derived mesenchymal stem cells cultured in FBS and PL supplements.

Cell Cycle Phase (%)	SubG1	G0G1	S	M
Explant FBS	3.6 ± 0.4	84.6 ± 3.0	5.9 ± 0.8	5.7 ± 1.7
Explant PL	3.4 ± 1.1	75.2 ± 8.4	8.4 ± 2.8	12.8 ± 5.2
Enzymatic FBS	2.6 ± 0.6	81.0 ± 5.1	6.9 ± 1.9	9.3 ± 3.1
Enzymatic PL	4.0 ± 0.4*	71.2 ± 7.7	8.65 ± 1.2	16.0 ± 6.7

Data presented as mean percentage ± SEM of three independent experiments. Statistical comparisons were made within explant groups and within enzymatic groups. *P < 0.05.

### PL supplementation does not alter immunomodulatory effects of MSC

**Figure 6:**
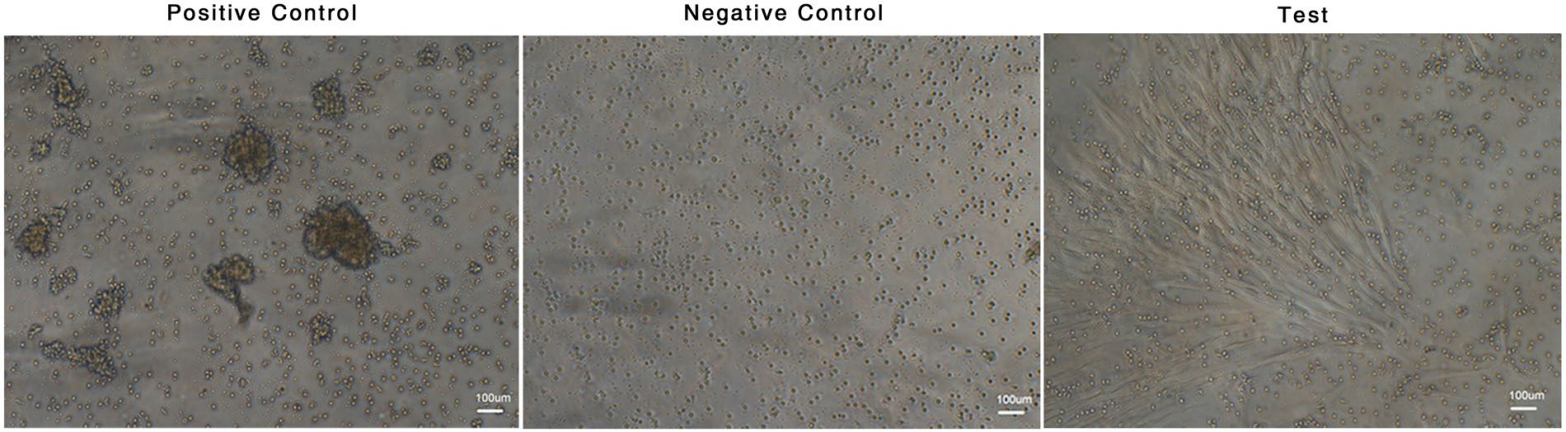
Immune suppression assay by mixed lymphocyte reaction. Representative bright-field images of positive control (PBMNC with PHA) showing aggregation of cells indicating proliferation of lymphocytes, negative control (PBMNC without PHA) showing no aggregation stating for the absence of proliferation of lymphocytes and PL expanded MSCs co-cultured with allogeneic PBMNC in the presence of PHA showing considerably less aggregates indicative of immunomodulation.

Mixed Lymphocyte Reaction (MLR) measures the rate of proliferation of PHA stimulated allogeneic lymphocytes co-cultured with MSC. A standard MLR assay was set up by co-culturing the CFSE labelled PBMCs over the overnight adhered MSC. Proliferation of lymphocytes was visualized under bright field microscope and analyzed by flow cytometry. Figure 6 shows the bright-field images of cell aggregates/clumps indicating the proliferation of T cells, following PHA stimulus in positive control. No cell aggregates were seen in negative control under unstimulated condition and relatively very few cell aggregates were seen in MSCs co-cultured with stimulated lymphocytes. MSCs exerted immunosuppression on peripheral blood lymphocytes from allogeneic donors by inhibiting the lymphocytes proliferation in response to PHA *in vitro*.

**Figure 7:**
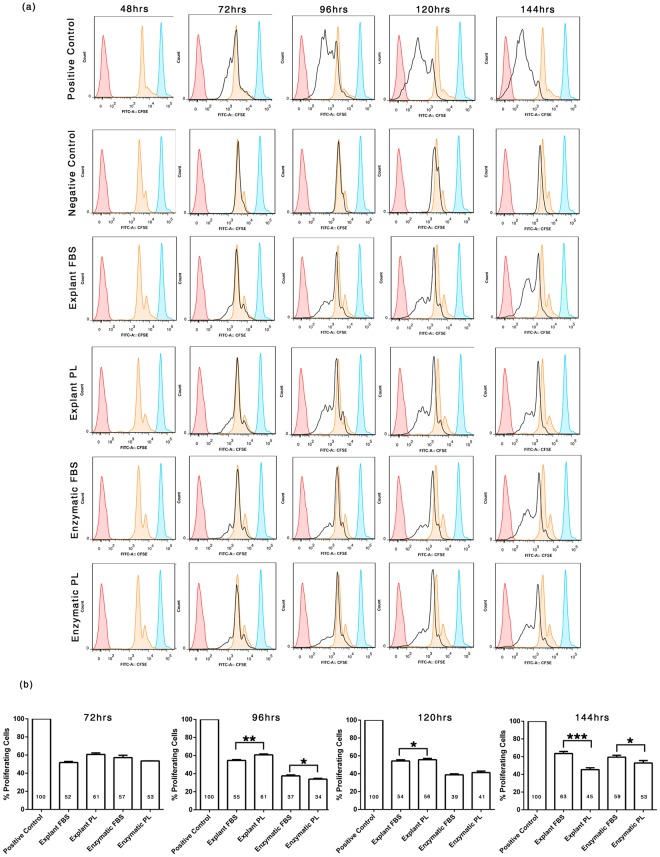
CFSE-based lymphocyte proliferation assay. (**a**) Flow cytometry analysis exhibited several histograms representing unstained lymphocytes at 0 h (represented by pink histogram), CFSE stained lymphocytes at 0 h (represented by blue histogram), CFSE dilution peak (represented by orange histogram) and proliferation of stained lymphocytes with respect to CFSE expression level (represented by black histogram). When the lymphocytes proliferate, new peaks moving towards the left of the initial peak with reduced dye intensity is seen. (**b**) Percentage of proliferating lymphocytes co-cultured with MSCs were normalized with control from the CFSE dilution peak. Statistical comparisons were made within the explant and enzymatic groups respectively over different time points using student’s t test. *P < 0.05, **P < 0.01, ***P < 0.005.

Flow cytometry analysis generated histograms, revealing the distribution of cells relative to CFSE expression level (Fig. 7a). A CFSE dilution profile at 48 h, termed as first-generation peak (orange histogram in Fig. 7a) was obtained following PHA stimulus and mitotic division of T lymphocytes. When the cell divides, there is an equal distribution of dye to the progeny cells thus reducing the fluorescence to half in the daughter cells. In the positive control, PHA stimulated lymphocytes proliferated showing an increase in number of daughter peaks with gradual shift towards the left from the first-generation peak over a period of 72, 96, 120 & 144 h demonstrating the dilution of dye due to CFSE distribution between daughter cells following cell division (black histogram in Fig. 7a). In the negative control, unstimulated lymphocytes did not proliferate and flow cytometry generated a histogram where the lymphocyte overlaid the first-generation peak and did not shift to the left up to 144 h (black histogram in Fig. 7a). PHA stimulated lymphocytes co-cultured with explant-MSC expanded in FBS generated a histogram where there was only a marginal shift towards the left with most of the daughter peaks residing in the same region of first-generation peak up to 144 h. This indicates reduction in lymphocyte proliferation even with PHA stimulation, that is in turn suggestive of immunomodulation by MSCs. Qualitatively the equivalent peaks and shift in PL supplemented explant MSC are further reduced indicative of improved immunomodulation by MSCs in PL. The histograms of enzymatically dissociated MSC supplemented with FBS and PL co-cultured with lymphocytes exhibited immunomodulation with no differences in peaks and shifts as observed in explant cultures. This suggest that PL may improve immunomodulation when coupled with explant cultures.

Furthermore, the percentage of lymphocyte proliferation was calculated by normalizing to control from the CFSE dilution peak (Fig. 7b, Table 3). Proliferation of lymphocytes in the absence of MSCs was assumed as 100% proliferation (control). The percentage of proliferating lymphocytes in explant derived MSC cultures with FBS were 54% at 72 h, 55% at 96 h, 54% at 120 h and 64% at 144 h, whereas in explant derived MSC cultures with PL were 61% at 72 h, 61% at 96 h, 56% at 120 h and 45% at 144 h respectively demonstrating superior immunosuppressive property with reduction in lymphocyte proliferation in MSC expanded with PL at 144 h over MSC expanded with FBS by explant cultures. The percentage of proliferating lymphocytes in enzymatic derived MSC cultures with FBS were 57% at 72 h, 38% at 96 h, 39% at 120 h and 59% at 144 h, whereas in enzymatically derived MSC cultures with PL were 53% at 72 h, 34% at 96 h, 41% at 120 h and 53% at 144 h respectively demonstrating significant reduction in lymphocyte proliferation co-cultured with MSC expanded with PL at 144 h.

**Table 3:** Immunosuppressive effect of umbilical cord tissue derived mesenchymal stem cells cultured in FBS and PL supplements on lymphocyte proliferation assessed by MLR assay.

% Proliferating Lymphocytes	72 h	96 h	120 h	144 h
Explant FBS	51.78 ± 1.2	54.65 ± 0.9	54.25 ± 1.3	63.56 ± 2.3
Explant PL	60.70 ± 1.6	60.76 ± 1.0**	55.66 ± 1.4*	45.35 ± 2.1***
Enzymatic FBS	57.20 ± 2.5	37.56 ± 1.0	38.81 ± 1.0	59.44 ± 2.0
Enzymatic PL	53.52 ± 0.1	33.95 ± 0.8*	41.27 ± 1.6	52.84 ± 2.7*

Data presented as mean percentage ± SEM of two independent experiments. Statistical comparisons were made within explant and enzymatic groups respectively. *P < 0.05, **P < 0.01, ***P < 0.005.

### PL supplementation maintains the chromosomal stability of MSC

**Figure 8:**
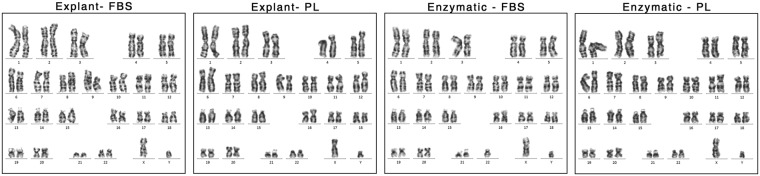
Cytogenetic analysis. Normal Q-banding karyotype obtained from at least 20 proliferating cells of FBS and PL cultured MSC preparations of Passage 4 analyzed revealed a normal 46XY karyotype.

To confirm that long term supplementation of MSC cultures with PL for chromosomal stability, we examined chromosomes of at least 20 proliferating cells per line as per the guidelines of the international system for human cytogenetic nomenclature recommendations. PL and FBS supplemented MSC cultures of explants and enzymatic dissociated MSCs showed a normal diploid karyotype (46XY chromosomes) with no gross abnormalities (Fig. 8).

## Discussion

MSCs have been derived from many different tissue sources^25^, however the use of human umbilical cord tissue derived mesenchymal stem cells have gained considerable attention over other origins due to the young donor age, non-invasive procurement, ample starting material, exhibition of shorter proliferation time and display of higher expansion potential making them an appealing candidate in tissue engineering and cell therapy^26^. The increasing number of clinical trials using MSC to treat diseases and other pathological conditions have led to the requirement of a replacement for FBS that is xeno-free and allogeneic as recently recommended by the US-FDA^10^. Successful outcomes of clinical applications are highly correlated with sufficient numbers of transplanted MSCs at early passage. The FDA suggests that this approach increases potency of MSCs and reduces the immune reactions in cellular therapy^27^. Thus, the xeno-free and allogeneic supplement should also support high yield at low passage number of primary cultures of MSCs reducing long term extended cultures of expansion^9^. Viral transmission from animal serum has been reported^28^ and antigens in FBS (such as bovine antigen) have been shown to induce immunological responses in recipients. It has also been shown that *in vitro* culture expanded MSCs internalizes bovine protein antigen with FBS supplement approx. in the range of 7–30 mg for every 100 million cultured MSCs^12^. The variability in commercially available serum free medium has led to inconsistent results in proliferation and MSC characteristics^29,30^. Human PL addresses all these concerns and is a highly feasible alternative to FBS as demonstrated by earlier studies^31,32^. Fibrin gel clot formation however has to be prevented or neutralized for PL to be usable in culture. Presence of plasma coagulation factors and fibrinogen in the PL tends to form fibrin clots in response to calcium present in basal medium (α-MEM), while preparing PL supplemented medium. Under such instances, mechanical depletion of fibrin clots by disrupting the hydrogel formation and by removing the fibrin pellet upon centrifugation of PL supplemented medium is a safe and efficient alternative to commercially available heparin which is of porcine origin. Additionally usage of heparin, has been shown to negatively affect proliferation, differentiation and migration of MSC^33^.

PL showed competency in maintaining MSC characteristics as defined by the ISCT such as plastic adherence, surface marker expression and tri-lineage differentiation potential^34^. Interestingly, explant culture of UCT showed increased yield of viable cells with 10% PL compared to 20% FBS with no notable difference in cell yield from the enzymatically dissociated cultures, corroborating studies that demonstrated accelerated yield of harvest and expansion of cells at P0 by supplementing the medium with 10% PL^35^. Explant cultures have been shown to preserve the ECM intact, be under less mechanical stress, and harbor homogenous population of cells^36^. We speculate that the increase in viable cell yield of potent MSC from explant cultures may be the result of the cellular micro-environment responding to factors in the PL which in turn stimulate MSC migration and expansion. The enzymatically dissociated cultures could lack such micro-environmental cues or the supporting cells due to increased manipulation of tissue which could in turn explain the even cell yield between PL and FBS cultures^37^. Explant cultures are simple, cost-effective and more efficient than enzymatic dissociation for harvesting MSC from hUCT. In addition the enzymatic digestion of tissue increases the risk of contamination with increasing processing time. Therefore, coupling explant MSC cultures with PL can maximize the yield of early passage potent MSC (as described earlier) for clinical applications^27^. In addition, the concentration of PL used is half the concentration of FBS throughout the culture strategy in this study. We compared 10% PL with 20% FBS as these concentrations have been employed for harvest of MSC from hUCT. Furthermore, subsequent passages employed only 5% PL for culture. Furthermore, subsequent passages for the *in vitro* expansion of MSCs employed only 10% FBS and 5% PL. The reduced concentration of PL supplementation allows for the maintenance of original microenvironment in primary and expanded cultures by reducing excessive exposure to cytokines and other stimulatory factors. There was also no change in cell cycle phases as flow cytometry revealed that majority of cells of PL supplemented cultures resided in G0/G1 phase indicative of a healthy metabolically active state equivalent to MSC cultured in FBS. It is imperative that a new supplementation alternative such as PL should not introduce any chromosomal instability that may increase the chances of malignant transformation, or decrease immunosuppressive, regenerative and reparative characteristic of MSCs. Chromosomal instability usually leads to alterations in MSC function and characteristics. Karyotyping analysis revealed for no gross chromosomal instability in PL supplemented MSC.

Selective suppression of the immune system is a characteristic feature of MSC that prevents GVHD during allogeneic stem cell transplantation^38^. MSCs are immune privileged cells due to the low expression of Class II major histocompatibility complex (MHC-II) and absence of co-stimulatory molecules such as CD80, CD86 and CD40 on their surface. Immunomodulation is largely mediated by paracrine factors such as IFN-γ, TNF-α, IL-1β, and IL-2 secreted by MSCs which efficiently inhibit T cell proliferation^39^. The MLR assay, tests for the immunomodulation by MSC on allogeneic T cells *in vitro* and is considered as the most appropriate functional assay for the use of MSC in immunotherapy^40^. Our results showed that explant and enzymatically cultures derived MSCs expanded using PL had the superior capacity to suppress the proliferation of allogeneic lymphocytes over a long time of 144 h. MSCs cultured in PL inhibited the proliferation of lymphocytes at a relatively high concentration of 1:10 in response to PHA confirming that PL supplementation does not affect the immunomodulation property of MSC *in vitro*. Further *in vivo* studies and clinical trails are needed to confirm for immunosuppressive property using MSC expanded with PL supplement.

## Materials and Methods

### Human Subjects

This study was approved by the Institutional ethics committee (IEC) of The Indian Institute of Technology Madras, Chennai, India (IEC/2018/01/RSV-5/10) and the work with human samples was carried out in accordance with the Helsinki Declaration. Human umbilical cord tissue samples (n = 3) were collected from caesarean deliveries to avoid the risk of contamination^41^. All samples were collected at Chennai managaratchi magaperu maruthuvamanai government hospital, Kodambakkam, Chennai, India, with written informed consents from the delivering mothers. Samples were transported to the laboratory in 1X PBS (Gibco, USA) containing antibiotics within 4 hours of collection at 4 °C.

### Preparation of Pooled Human Platelet Lysate (pHPL)

Expired platelet units of blood group O and AB, negative for infectious diseases were obtained from the blood banks. Platelet count was enumerated and batch of pHPL with a platelet count of ≥2 × 10^8^ cells/mL was employed in this study. Platelet units were subjected to three freeze-thaw cycles and pooled at the ratio of 3:1 (Blood group O: AB) to counter the effects of ABH antigens and isoagglutinins^42^. Pooled platelets were centrifuged at 4000 × g for 15 minutes and the pellet of platelet fragments and cell debris was discarded. Supernatant was strained through a 40μm cell strainer and further filtered through a 0.22 μm PES filter (Merck Millipore, Billerica, MA, USA). Filtered product was stored as aliquots of 10 ml at −80 °C (Fig. 1b).

### Preparation of complete medium using pooled human Platelet lysate (pHPL)

Complete medium using pooled human platelet lysate were prepared by mechanical depletion of fibrin gel clot formation^33^. α-MEM (PAN Biotech, GmbH) supplemented with 10% pHPL/5% pHPL and antibiotics was prepared and incubated for 4 h at room temperature followed by overnight incubation at 4 °C for hydrogel formation. The coagulated medium was then incubated at 37 °C for 1 h to allow complete fibrin clotting. The medium was centrifuged at 4000x g for 10 minutes at RT without disturbing the pellet. The resulting clear medium supernatant was filtered through a 0.22 µm PES filter and stored at 4 °C for further use.

### Processing and expansion of MSCs from human umbilical cord tissue

The human umbilical cord tissues were processed within 12 h of sample collection by the two most commonly used approaches as schematically outlined in Fig. 2a. Cords were washed in 1X PBS containing antibiotics thrice to remove the blood clots. For the explant method, cord tissue was minced to approx. 0.5 cm cubes and placed in 0.1% gelatin coated 6 well plates with α-MEM medium supplemented with 20% FBS (Gibco) and 10% PL respectively along with antibiotics. For the enzymatic methodology, cord tissue was minced finely and digested for 4–5 h using collagenase type I (Worthington Biochemical Corporation) at 1 mg/ml. Digested mixtures were then washed and cultured with α-MEM medium supplemented with 20% FBS and 10% PL respectively along with antibiotics onto a 0.1% gelatin coated 6 well plates. Cultures were incubated in a humidified incubator at 37 °C with 5% CO_2_. Medium for explant cultures were changed on day 3, day 10 and every 3^rd^ day till the day 20. For enzymatic cultures, medium was changed after 48 hours and every 3^rd^ day until day 20. Microscopic examination for confluence of cultures and morphology of cells was carried out at regular intervals. Viable cell numbers from primary cultures were enumerated by trypan blue dye exclusion. Further expansion of these MSCs through subsequent passages was carried out using 5% PL and 10% FBS respectively on 0.1% gelatin coated tissue culture flasks.

### Immunophenotypic analysis

The expression of MSC surface markers were assessed by flow cytometry analysis. Cells were trypsinized into a single cell suspension and incubated with the fluorochrome-conjugated antibodies against human antigens, including anti-CD34-PE, anti-CD45-FITC, anti-CD90-FITC (BD Biosciences), anti-CD73-FITC (Bio Legend) and anti-CD105-FITC (R&D systems) for 15 mins in the dark at RT. After incubation, cells were washed with 1X PBS, centrifuged at 1500 rpm for 5 minutes and suspended in 1X PBS for flow cytometry analysis. Samples were acquired on a FACS canto II flow cytometer (BD Biosciences) and data were analyzed using FlowJo software (Tree Star).

### Tri-lineage Differentiation Assay

#### Adipogenesis

PL and FBS supplemented MSCs at P3 were seeded onto 0.1% gelatin coated 6-well plates at 4000 cells/cm^2^ and grown at 37 °C in a humidified atmosphere with 5% CO_2_ until 70–80% confluence was reached. Cultures were then induced with adipogenesis inducing medium (AIM) (STEMPRO^®^ Adipogenesis differentiation kit, GIBCO, USA) for 14 days with medium replacement every 3 days. Lipid droplet accumulation was visualized using bright field microscope after fixing the cells with 4% paraformaldehyde (PFA) and staining with oil red O (Sigma, USA).

#### Osteogenesis

PL and FBS supplemented MSCs at P3 were seeded onto 0.1% gelatin coated 6-well plates at 4000 cells/cm^2^ and grown at 37 °C in a humidified atmosphere with 5% CO_2_ until 70–80% confluence was reached. Cultures were then induced with osteogenesis inducing medium (OIM) (STEMPRO^®^ Osteogenesis differentiation kit, GIBCO, USA) for 21 days with medium replacement every 3 days. Mineral deposition was visualized using bright field microscope after fixing the cells with 4% PFA and staining with alizarin red S (HiMedia, India).

#### Chondrogenesis

PL and FBS supplemented MSCs at P3 were seeded onto 0.1% gelatin coated 6-well plates as 5 μL droplets with 0.1 × 10^6^ cell per droplet to generate micromass culture. Cells were incubated at 37 °C in a humidified atmosphere with 5% CO_2_ for 2 h. On adherence, micromass cultures were induced with chondrogenesis inducing medium (CIM) (STEMPRO^®^ Chondrogenesis differentiation kit, GIBCO, USA) for 14 days with medium replacement every 3 days. Presence of glycosaminoglycan, was visualized using a bright field microscope after fixing the cells with 4% PFA and staining with safranin O (HiMedia, India).

### Growth curve and Cell Cycle

PL and FBS supplemented MSCs at P3 obtained from explant cultures were cultured in 0.1% gelatin coated T25 Flasks at 4000cells/cm^2^. Cells were harvested and viable cell numbers were enumerated daily up to 5 days by trypan blue dye exclusion. Growth curve was plotted according to number of the cells and population doubling time (PDT) was calculated from the growth curve.

Cell cycle analysis of MSC cultured in FBS and PL was performed using propidium iodide staining (Sigma Aldrich, USA). MSC cultures were synchronized by starving them for 24 h after attaining 50% confluence. MSC cultures were then replenished with complete medium for 24 h. 1 × 10^5^ MSCs in the exponential growing phase were collected and fixed with ice-cold 70% ethanol at 4 °C for 24 h. Cells were then stained with staining solution comprising of 50 μg/ml propidium iodide, 50 µg/ml RNase A (MP Biomedicals, USA) and 0.1% Tritox X-100 (Sigma-Aldrich) and incubated at 37 °C for 30 min at dark. Cell cycle phases were detected by FACS canto II flow cytometer (BD Biosciences) and the results were analyzed by FlowJo software (Tree Star).

### Lymphocyte Proliferation Assay

The immunoregulatory effect of MSCs at P3 cultured in PL and FBS supplements were evaluated by co-culturing MSCs with carboxyfluorescein diacetate-succinimidyl ester (CFSE) labelled peripheral blood mononuclear cells (PBMNCs)^43^. MSCs were plated onto flat-bottomed 96 well plates with 1.25 × 10^4^ cells/well in 200μl of RPMI 1640 (Gibco, USA) supplemented with 2 mM glutamine (Gibco, USA), 10% FBS/5% PL, and antibiotics. Culture plate was incubated in humidified atmosphere at 37 °C in 5% CO_2_ overnight allowing the cells to adhere. Human PBMNCs isolated from heparinized blood were obtained from healthy donors by density gradient centrifugation using Ficoll-Paque PLUS (GE Healthcare) at 400x g, for 30 min at RT. PBMNCs were fluorescently-labelled with 2 μm CFSE (Invitrogen, USA) by incubating for 15 mins in the dark. Five volumes of ice-cold RPMI 1640 supplemented with 10% FBS was added and incubated for 5 minutes in the dark. Cells were then washed and 1.25 × 10^5^ CFSE-stained PBMCs were added to the wells previously seeded with 1.25 × 10^4^ adherent MSCs to obtain a 1:10 MSCs to PBMCs ratio. Co-cultures were incubated with 0.25% phytohaemagglutinin (PHA) to stimulate T lymphocytes. PBMC cultures without MSCs were used as controls. Positive controls were treated with PHA while negative controls were left untreated. Cells were cultured for 6 days in a humidified atmosphere at 37 °C with 5% CO_2_. Aliquots of CFSE-labelled cells co-cultured with MSCs along with controls were harvested at different time points of 48, 72, 96, 120 and 144 h, and acquired on a BD FACS Canto II flow cytometer.

### Karyotype analysis

Chromosomes of at least 20 proliferating cells per line at P4 were counted and fully analyzed using G-banding at Human Genetics Department, Sri Ramachandra Medical College and Research Institute, Chennai, India.

### Statistical analysis

Data was compared using paired one tailed student’s t test. Data was presented as arithmetic mean ± standard error of the mean (SEM). Analysis was done with GraphPad Prism 6 and significant results are assigned asterisks (*P < 0.05, **P < 0.01, ***P < 0.005).

### Conclusion and perspective for future research

To our knowledge this is the first report that compares the yield of MSC from primary cultures of hUCT through explant culture and enzymatic dissociated cultures, supplemented with hPL. We recommend the use of PL from expired platelets as a viable alternative to FBS for generating clinically significant numbers of explant derived MSCs for transfusion in cellular therapy. In conclusion, coupling of explant cultures of hUCT with PL supplementation offers superior cellular and functional properties in MSC. PL supplementation for MSC culture could be adopted by public and private stem cell banking sectors by establishing a network with blood banks for the re-use of biological waste such as expired platelets. PL is also an economically viable alternative to FBS with cost of production estimated to be approximately 3-fold lesser than commercially available FBS. Production of PL using good manufacturing practice (GMP) with appropriate regulatory guidelines for relevant parameters needs to be laid down according to criteria established by International society of blood transfusion following which PL could become the *de facto* supplementation for stem cell culture^44^. While PL may also suffer from batch to batch variability as is commonly associated with FBS, we speculate that the variation will be considerably less as platelet contents are uniform. However, further studies need to be carried out to understand the variations within platelet units such as platelet count, donor age and concentration of various growth factors which are released on lysis and are anticipated to impact MSC harvest and expansion^45,46^.
